# Impaired Surface Expression of HLA-DR, TLR2, TLR4, and TLR9 in Ex Vivo-In Vitro Stimulated Monocytes from Severely Injured Trauma Patients

**DOI:** 10.1155/2017/2608349

**Published:** 2017-02-01

**Authors:** David Heftrig, Ramona Sturm, Elsie Oppermann, Kerstin Kontradowitz, Katrin Jurida, Lukas Schimunek, Mathias Woschek, Ingo Marzi, Borna Relja

**Affiliations:** ^1^Department of Trauma, Hand and Reconstructive Surgery, Goethe University, Frankfurt, Germany; ^2^Department of General and Visceral Surgery, Goethe University, Frankfurt, Germany

## Abstract

*Objective*. Trauma patients (TP) frequently develop an imbalanced immune response that often causes infectious postinjury complications. Monocytes show a diminished capability of both producing proinflammatory cytokines and antigen presentation after trauma. TLR2, TLR4, and TLR9 recognize pathogens and subsequently activate monocytes. While there are conflictive data about TLR2 and TLR4 expression after trauma, no studies about the expression of TLR2, TLR4, TLR9, and HLA-DR on monocytes from TP after their secondary ex vivo-in vitro “hit” have been reported.* Methods/Results*. Ex vivo-in vitro lipopolysaccharide- (LPS-) stimulated blood from TP showed diminished interleukin- (IL-) 1*β*-release in TP for five postinjury days compared to healthy volunteers (HV). The recovery was observed at day 5. In parallel, monocytes from TP showed an impaired capability of TLR2, TLR4, and TLR9 expression after secondary stimulation compared to HV, while the measurement of unstimulated samples showed significant reduction of TLR4 and TLR9 at ED. Furthermore, HLA-DR decreased after trauma and was even more profound by stimulation of monocytes. Ratio of monocytes to leukocytes was significantly increased at days 6 and 7 after trauma compared to HV.* Conclusion*. Impaired expression of TLRs and HLA-DR in acute inflammatory conditions may be responsible for the well-described monocyte paralysis after severe trauma.

## 1. Introduction

Despite recent significant improvements with regard to the treatment of severely injured trauma patients (TP), clinical complications, which develop due to increased susceptibility to opportunistic infections, are often [1, 2]. Trauma patients constitute a highly heterogeneous cohort of patients regarding their immune response. However, sepsis and multiorgan failures (MOF) are still predominant causes of late mortality after trauma [2, 3].

In response to injury, the innate and adaptive immune system is activated. The release of damage-associated molecular patterns (DAMPs) caused by tissue damage induces a systemic inflammatory response that attempts to neutralize the pathogen microorganisms and initiate the tissue repair mechanisms [4]. This process is defined as the systemic inflammatory response syndrome (SIRS) [5]. In parallel, trauma causes a counterbalancing immune response, so-called compensatory anti-inflammatory response syndrome (CARS) [6, 7]. The simultaneous development of SIRS and CARS results in a mixed antagonist response syndrome (MARS) [7]. Therefore, intensive care patients who initially survive the trauma impact may still undergo a persistent inflammation, immunosuppression, and catabolism syndrome (PICS) [8].

As critical regulators of the immune system, human monocytes exert a decreased capability of releasing proinflammatory cytokines such as tumor necrosis factor-alpha (TNF-alpha) and interleukin-1-beta (IL-1*β*) after a secondary ex vivo-in vitro exposure to endotoxin after trauma [9–11]. This has been illustrated on postinjury days 2, 5, and 10 [12]. On the other hand, other studies described a monocytosis after trauma and surgical sepsis [13, 14]. Interestingly, expression of the major histocompatibility complex 2 (MHC 2) and activator of the T-cell receptor human leukocyte antigen-DR (HLA-DR) decreased on monocyte's surface (mHLA-DR) after trauma [15, 16]. Persisting low levels of mHLA-DR have been associated with major sepsis development, while patients with uneventful recovery reached normal levels of mHLA-DR after trauma [15, 17]. The decreased immune response to tissue injury carries the potential for immunological dysfunctions, which often cause infections and/or multiorgan disorders in trauma patients [18].

DAMPs arise from tissue damage and are recognized by leukocytes* via* their pathogen recognition receptors (PRRs) [19]. The toll-like receptors (TLRs, in mammalian 13 different types) are specific subtypes of PRRs [20, 21]. TLRs play an important role in the intracellular signalling and subsequent induction of the innate immunity [22]. TLR2 is activated by different ligands such as peptidoglycan and lipoteichoic acid deriving from Gram-positive microorganisms [23, 24]. TLR4 recognizes endotoxins of Gram-negative bacteria (e.g., lipopolysaccharide (LPS)) [25]. Together with CD14 and the LPS-binding protein (LBP), TLR4 activates monocytes and subsequently increases both transcription and the release of proinflammatory cytokines [26, 27]. By recognizing of bacterial DNA with high amounts of unmethylated CpG dinucleotides TLR9 activates the immune response [28, 29]. In several studies, TLR9 is described as an intracellular receptor; however, there are reports describing its surface expression as well [30–33]. Compared to control subjects, trauma patients exert an impaired capability to produce proinflammatory cytokines after CpG-oligonucleotide stimulation [34]. Different expression patterns of TLRs on circulating monocytes have been reported after trauma. Pérez-Bárcena et al. have postulated higher expressions of TLR2 and TLR4 [35], while others detected reduced TLR4 expression, with unchanged TLR2 [34], reduced TLR2 expression with unchanged TLR4 expression [36], and reduced expression of both TLR2 and TLR4 in TP [37]. Taken together, these studies do not provide an explanation for the diminished cytokine release and impaired activity of monocytes after trauma. Furthermore, the expression analysis of TLRs after a secondary ex vivo-in vitro stimulation as described in this study has never been performed before in trauma patients.

Here, the expression and coexpression of TLR2, TLR4, TLR9, and of HLA-DR were measured on circulating CD14 positive monocytes after severe trauma. In order to simulate secondary acute inflammatory conditions after trauma, the samples were stimulated with a leukocyte activation cocktail (LAC) and the (co-)expression of these receptors was evaluated. In addition, a daily analysis of the IL-1*β* releasing capability of patient's monocytes after their LPS stimulation was conducted until posttrauma day 10.

## 2. Patients and Methods

### 2.1. Ethics

The study was performed in the University Hospital Frankfurt, Goethe University, Germany, with the institutional ethical committee approval (312/10) in accordance with the Declaration of Helsinki and following STROBE guidelines [38]. All patients or their legally authorized representative as well as included healthy volunteers signed the written informed consent form.

### 2.2. Patients

In our prospective clinical experimental trial, 29 severely injured trauma patients with a history of acute blunt or penetrating trauma and an injury severity score (ISS) ≥ 16 were enrolled. Exclusion criteria were being younger than 18 or older than 80 years of age, severe burn injury, acute myocardial stroke, cancer or chemotherapy, immunosuppressive drug therapy, HIV, infectious hepatitis, acute CMV infection, and/or thromboembolic events. Upon arrival at the emergency department (ED), vital signs were measured and the ISS was calculated according to the abbreviated injury scale (AIS) as of 2008 [39, 40]. The control group consisted of 14 healthy volunteers (HV).

### 2.3. Blood Sampling

Blood samples were withdrawn in ethylenediaminetetraacetic acid (EDTA) tubes (Sarstedt, Nürmbrecht, Germany) directly after admission to the ED and daily until day 10 after trauma. The samples were kept either at room temperature for functional assays or on ice for flow cytometric analysis. The subsequent blood samples taken daily from TP as well as blood samples from HV were obtained between 7 and 11 a.m.

### 2.4. Ex Vivo-In Vitro Whole Blood Stimulation for Cytokine Production Assay

Blood samples (50 *μ*l) were diluted in 450 *μ*l RPMI 1640 (Seromed, Berlin, Germany; polypropylene tube, BD Bioscience, Franklin Lakes, NJ, USA) supplemented with 10% heat-inactivated fetal calf serum (FCS), 100 IU/mL penicillin, and 100 *μ*g/mL streptomycin (Gibco, Karlsruhe, Germany) and 20 mM HEPES buffer (Sigma, Deisenhofen, Germany). The samples were stimulated with LPS (5 *μ*g/ml,* E. coli* 0111:B4, SIGMA-Aldrich) and incubated at 37°C and 5% CO_2_. Twenty-four h later, the samples were centrifuged at 2100*g* for 15 minutes, and the supernatant was collected and stored at −80°C until assay. To address unspecific stimulation, corresponding blood samples were incubated as described above without LPS stimulation.

The samples were assayed for IL-1*β* using the enzyme-linked immunosorbent assay (ELISA) technique (Quantikine®, Human IL-1*β*/IL-1F2 Immunoassay ELISA, R&D Systems) according to the manufacturer's instructions.

### 2.5. Ex Vivo-In Vitro Whole Blood Stimulation for Cell Surface Receptor Analysis

Blood samples (100 *μ*l) were diluted in 395 *μ*l RPMI 1640, and 5 *μ*l of the leukocyte activation cocktail (LAC, containing the phorbol ester, phorbol 12-myristate 13-acetate (PMA), a calcium ionophore (Ionomycin), and the protein transport inhibitor BD GolgiPlug™ (Brefeldin A), BD Pharmingen™) were added. The samples were incubated for 5 h at 37°C under 5% CO_2_ and stained for flow cytometric analysis as described below. Unstimulated corresponding samples were evaluated as controls.

### 2.6. Measurement of Cell Surface Receptor Expression by Flow Cytometry

**Figure 1:**
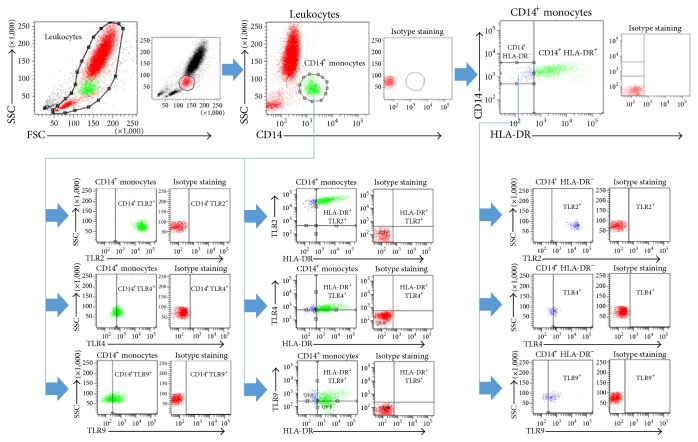
Gating strategy for the flow cytometric analysis and evaluation.

Blood samples (100 *μ*l) were transferred into polystyrene FACS tubes (BD Pharmingen) and incubated with mouse anti-human CD14 V500 (Clone M5E2, BD Bioscience, San Jose, CA), mouse anti-human TLR2 PE-Cy7 (Clone T2.5, eBioscience, San Diego, CA), mouse anti-human TLR4 FITC (Clone HTA125, IMGENEX, San Diego, CA), mouse anti-human TLR9 Alexa Flour® 647 (Clone 26C593.2, IMGENEX, San Diego, CA), and mouse anti-human HLA-DR PerCP-Cy5.5 conjugated (Clone L243, BD Bioscience, San Jose, CA) antibodies. After 30 minutes at room temperature, 3 ml of FACS lysing solution (FACS Lysing Solution, BD Pharmingen) was added for additional 10 minutes. Subsequently, the samples were centrifuged at 400 g for 5 minutes and washed twice with 4 ml phosphate-buffered saline (PBS) supplemented with 0.5% bovine serum albumin (FACS buffer). Immediately after the supernatants were removed, the cells were diluted in 300 *μ*l FACS buffer and were then subjected to flow cytometry with a* BD FACS Canto 2*™ using FACD DIVA™ software (BD). The monocyte population was defined by gating CD14^+^ cells in the corresponding forward and side scatter scan. From each sample, a minimum of 20.000 monocytes was measured. The number of totally gated cells for each was calculated as absolute cell number in percentage relative to the ratio of the indicated cell populations in representative figures. The gating strategy is shown in Figure 1.

### 2.7. Statistical Analysis

GraphPad Prism 6.0 software (GraphPad Software Inc., San Diego, CA) was used to perform the statistical analysis. Data are given as mean ± standard error of the mean (SEM) or as absolute cell numbers calculated in percent. Student's *t*-test with Welch correction and one-way analysis of variance (ANOVA) with a Dunn post hoc test were used for comparison among all different groups. A *p* value below 0.05 was considered statistically significant.

## 3. Results

### 3.1. Study Population

29 patients with major trauma (TP) and 14 HV were enrolled in this study. The majority of the study subjects were male (TP: 76% versus HV: 64%). The mean age of TP was 46 ± 3 versus 37 ± 6 in HV. All patients were substantially injured (ISS: 28 ± 2). The mean stay in the intensive care unit (ICU) was 10 ± 2.0 days. The in-hospital stay duration was 20 ± 4 days. In moderate contrast to our previous studies [11, 41, 42], patients in this study represent the cohort of major trauma patients.

### 3.2. Time Course of the LPS Response in Whole Blood from Trauma Patients

**Figure 2:**
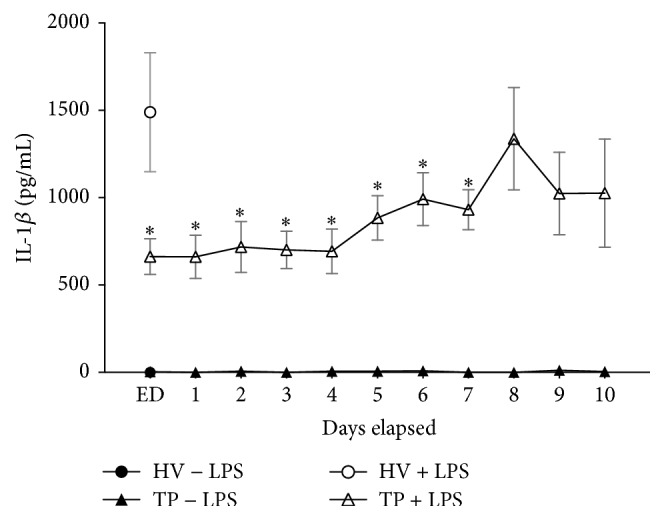
Major trauma leads to reduced IL-1*β* release from whole blood after LPS stimulation. Whole blood from healthy volunteers (HV, *n* = 14) or major trauma patients (TP, *n* = 29) was stimulated with LPS. Supernatants were collected after 24 h for measurements of IL-1*β* by ELISA. A 10-day time course after admission (emergency department, ED–10) was made. To address unspecific stimulation, corresponding blood samples were incubated as described above without LPS stimulation. Data are shown as mean ± SEM. ^*∗*^*p* < 0.05 versus LPS-stimulated HV.

Directly after admission, the IL-1*β* release was significantly decreased until day seven in TP compared to HV after LPS stimulation (ED: 663.0 ± 102.4 versus 1490.0 ± 340.5 pg/mL; *p* < 0.05, Figure 2). A trend to a continuous IL-1*β* secretion recovery in cells from TP was observed at day 5 after trauma. Even after 7 postinjury days, the IL-1*β* release was not recovered completely. Neither the nonstimulated samples of HV nor the nonstimulated samples of TP have shown any significant alterations in IL-1*β* release.

### 3.3. Ratio of CD14^+^ Monocytes to Leukocytes

**Figure 3:**
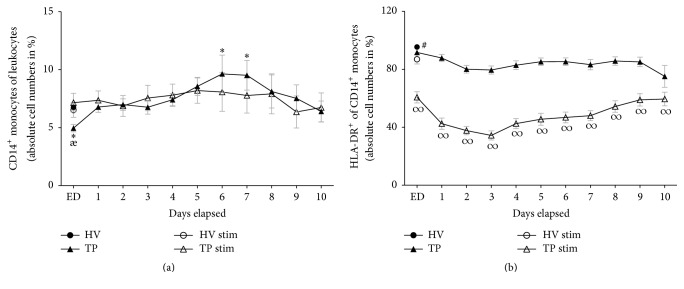
Whole blood from healthy volunteers (HV, *n* = 14) or major trauma patients (TP, *n* = 29) was analyzed by flow cytometry over a 10-day time course after admission (emergency department, ED–10). Monocytes were detected using anti-human CD14 in the corresponding sideward and forward scatter. Unstimulated (black symbols) and stimulated (clear symbols) measurements were made. For stimulation (stim), whole blood was incubated with leukocyte activation cocktail for 5 h with subsequent analyzing procedure as in unstimulated samples. Data are shown as mean ± SEM. (a) CD14^+^ monocytes to leukocytes ratio, (b) HLA-DR expression in CD14^+^ monocytes. ^*∗*^*p* < 0.05 versus unstimulated HV; ^*æ*^*p* < 0.05 TP versus corresponding TP stim; ^#^*p* < 0.05 HV ctrl versus all; ^*∞*^*p* < 0.05 TP stim versus corresponding unstimulated TP and HV stim.

TP had a significantly lower absolute cell numbers of CD14^+^ monocytes in total leukocyte population at ED compared to HV (4.95 ± 0.32 versus 6.76 ± 0.30 absolute cell number in %, *p* < 0.05, Figure 3(a)). Thus, on day 5, an increase in CD14^+^ monocytes was observed, with a significant maximum height on days 6 and 7 compared to HV (day 5: 8.56 ± 0.78; day 6: 9.64 ± 1.62; day 7: 9.51 ± 1.28 versus 6.76 ± 0.30 absolute cell number in %, *p* < 0.05, Figure 3(a)). At the end of observation period, the ratio of CD14^+^ monocytes to leukocytes in TP was comparable with HV. Stimulating whole blood samples with LAC did not markedly change the ratio of CD14^+^ monocytes to leucocytes in HV and TP (HV: 6.51 ± 0.63, Figure 3(a)).

### 3.4. Surface Expression of mHLA-DR on CD14^+^ Monocytes

During the whole observational period, the ratio of HLA-DR^+^ cells to total CD14^+^ monocytes remained significantly lower compared to HV (*p* < 0.05, Figure 3(b)). After the ex vivo-in vitro stimulation with LAC, HLA-DR expression on CD14^+^ monocytes from HV decreased significantly compared to unstimulated cells from HV (86.96 ± 3.28 versus 95.43 ± 1.35 absolute cell number in %, *p* < 0.05, Figure 3(b)). Stimulation of samples from TP reduced strongly the HLA-DR expression in CD14^+^ monocytes compared to both unstimulated samples from TP and stimulated samples from HV (ED: 60.68 ± 3.92 versus 91.67 ± 1.47 and 86.96 ± 3.28 absolute cell number in %, resp., *p* < 0.05, Figure 3(b)). The loss of surface HLA-DR expression after LAC stimulation in HV resulted in an 8.9% decrease, whereas the loss of surface HLA-DR expression in TP after LAC stimulation was significantly higher, that is, 33.8% in samples from ED (*p* < 0.05, Figure 3(b)).

### 3.5. Surface Expression of TLR2 on CD14^+^ Monocytes

**Figure 4:**
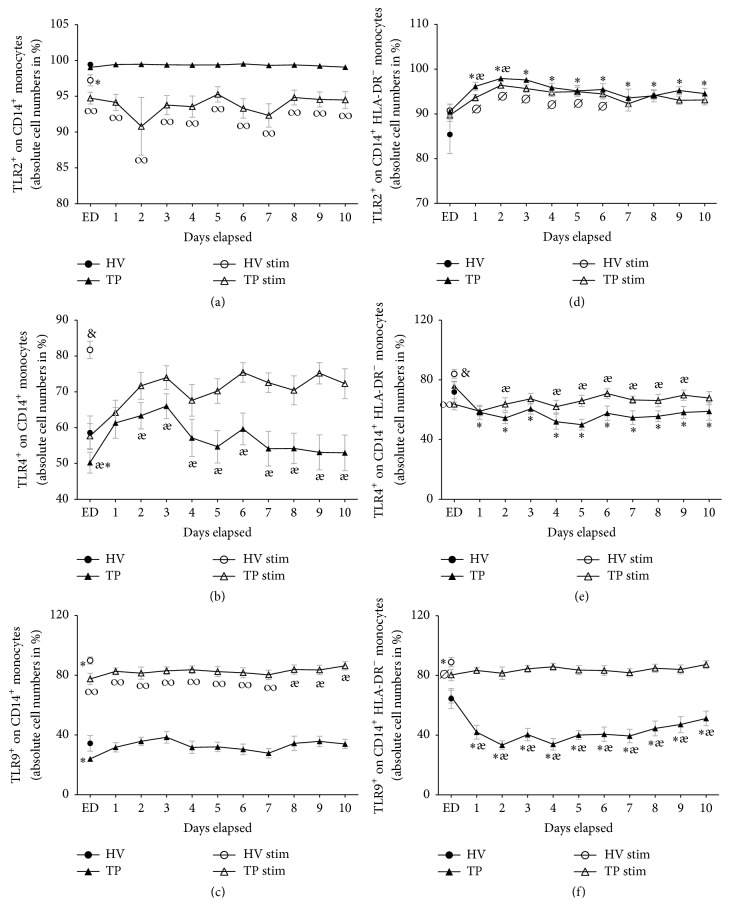
Whole blood from healthy volunteers (HV, *n* = 14) or major trauma patients (TP, *n* = 29) was analyzed over a 10-day time course after admission (emergency department, ED–10) by flow cytometry. Monocytes were detected using anti-human CD14 in the corresponding sideward and forward scatter. Unstimulated (black symbols) and stimulated (clear symbols) measurements were made. For stimulation (stim), whole blood was incubated with leukocyte activation cocktail for 5 h with subsequent analyzing procedure as in unstimulated samples. Data are shown as mean ± SEM. (a) TLR2 expression in CD14^+^ monocytes, (b) TLR4 expression in CD14^+^ monocytes, (c) TLR9 expression in CD14^+^ monocytes, (d) TLR2 expression in CD14^+^ HLA-DR^−^ monocytes, (e) TLR4 expression in CD14^+^ HLA-DR^−^ monocytes, and (f) TLR9 expression in CD14^+^ HLA-DR^−^ monocytes. ^*∗*^*p* < 0.05 versus unstimulated HV; ^*⌀*^*p* < 0.05 versus stimulated HV; ^*æ*^*p* < 0.05 TP versus corresponding TP stim; ^#^*p* < 0.05 HV ctrl versus all; ^&^*p* < 0.05 versus all; ^*∞*^*p* < 0.05 TP stim versus corresponding unstimulated TP and HV stim.

99.4% of all CD14^+^ monocytes from HV expressed TLR2. After trauma, that value did not change significantly (Figure 4(a)). LAC stimulation in samples from HV resulted in a significant decrease of TLR2 expression (97.2%, *p* < 0.05, Figure 4(a)). The TLR2 expression in CD14^+^ monocytes from TP was significantly decreased during the complete time course compared to unstimulated cells from TP as well compared to LAC-stimulated samples from HV (TP ED: 94.7 ± 0.8 versus 99.0 ± 0.2% and 97.2 ± 0.8%, resp., *p* < 0.05, Figure 4(a)).

### 3.6. Surface Expression of TLR4 on CD14^+^ Monocytes

58.6% of all CD14^+^ monocytes out of the HV group expressed TLR4. Directly after admission, the value was significantly lower in TP (50.2%, Figure 4(b)). During the observational period, it rose markedly beginning at day 1 (60.6 ± 4.1%) and showing no further significant changes during the posttraumatic time course compared to HV. LAC stimulation increased significantly the TLR4 expression in HV to 81.7% (*p* < 0.05, Figure 4(b)). The TLR4 expression in TP was significantly enhanced after LAC stimulation compared to unstimulated samples from TP (TP ED: 57.7 ± 3.5 versus 50.2 ± 2.9%, Figure 4(b)). Despite the continuously increased TLR4 expression on CD14^+^ monocytes in LAC-stimulated samples obtained from TP, the expression levels were still significantly lowered during the complete observational period compared to stimulated HV samples (Figure 4(b)).

### 3.7. Surface Expression of TLR9 on CD14^+^ Monocytes

34.4% of all CD14^+^ monocytes from HV expressed TLR9. Upon arrival to the ED, the value was significantly decreased to 24.0% (*p* < 0.05, Figure 4(c)). Nonetheless, the expression of TLR9 was comparable to the levels of HV after day 1. LAC stimulation increased significantly the TLR9 expression in HV and in TP during the whole observational period compared to unstimulated samples (HV: 89.9% versus 34.4%, TP ED 77.6 ± 3.8 versus 24.0 ± 1.6%, *p* < 0.05, Figure 4(c)). However, the levels of TLR9 expression after LAC stimulation were significantly lower until day 8 after trauma compared to HV (*p* < 0.05, Figure 4(c)). After day 8, TLR9 expression after LAC stimulation was comparable to stimulated HV samples.

### 3.8. Surface Expression of TLR2, TLR4, and TLR9 on HLA-DR^+^ or HLA-DR^−^ CD14^+^ Monocytes

#### 3.8.1. Surface Expression of TLR2 on HLA-DR^−^ and HLA-DR^+^ CD14^+^ Monocytes

85.4% of all CD14^+^ HLA-DR^−^ monocytes from HV expressed TLR2. Upon arrival to the ED, 90.5% of CD14^+^ HLA-DR^−^ monocytes from TP expressed TLR2, a value that was increased compared to HV, but this difference was not significant. During the subsequent whole posttraumatic observation period, the TLR2 expression was significantly increased compared to HV. LAC stimulation increased the TLR2 expression of HV to 90.7%; however, this increase was not significant (Figure 4(d)). Starting from day one until day six, LAC stimulation of samples from TP significantly increased the TLR2 expression compared to stimulated HV controls (*p* < 0.05, Figure 4(d)).

In contrast, the TLR2 expression on HLA-DR^+^ CD14^+^ monocytes showed no significant changes between HV and TP (HV: 99.7%, data not shown). Thus, LAC stimulation significantly decreased the TLR2 expression in TP during the complete study period in contrast to stimulated HV controls (data not shown).

#### 3.8.2. Surface Expression of TLR4 on HLA-DR^−^ and HLA-DR^+^ CD14^+^ Monocytes

71.9% of all HLA-DR^−^ CD14^+^ monocytes from HV expressed TLR4 (Figure 4(e)). Starting at day 1 until day 10, the TLR4 expression in HLA-DR^−^ CD14^+^ monocytes was significantly decreased in TP. LAC stimulation increased significantly the TLR4 expression to 84.0 in HV (*p* < 0.05, Figure 4(e)). During the whole posttraumatic observational period, the TLR4 expression after stimulation was significantly decreased in TP compared to stimulated HV.

The TLR4 expression in HLA-DR^+^ CD14^+^ monocytes was without significant differences in HV and TP (HV: 57.3%, data not shown). LAC stimulation of samples obtained from TP showed significantly decreased levels of the TLR4 expression on HLA-DR^+^ CD14^+^ monocytes at ED with no further significant changes in posttraumatic phase compared to stimulated samples from HV (HV: 74.0%, ED: 49.8%, *p* < 0.05, data not shown).

#### 3.8.3. Surface Expression of TLR9 on HLA-DR^−^ and HLA-DR^+^ CD14^+^ Monocytes

64.5% of all HLA-DR^−^ CD14^+^ monocytes from HV expressed TLR9. Starting at day 1, the TLR9 expression was significantly decreased in samples obtained from TP compared to HV for the whole time course (*p* < 0.05, Figure 4(f)). In HV LAC stimulation increased significantly the TLR9 expression to 88.9% (Figure 4(f)). The TLR9 expression in HLA-DR^−^ CD14^+^ monocytes after LAC stimulation was only significantly lowered in ED samples compared to simulated samples of HV. In the further study period, the value was comparable to stimulated samples of HV (Figure 4(f)).

The TLR9 expression in HLA-DR^+^ CD14^+^ monocytes was significantly decreased at the ED in TP (HV: 32.9%, ED: 20.2%, *p* < 0.05, data not shown); after day 1, the TLR9 expression was comparable to HV. However, the TLR9 expression in stimulated HLA-DR^+^ CD14^+^ monocytes of TP was significantly lower until day 8 after trauma compared to LAC-stimulated samples from HV (data not shown).

*TLR2, TLR4, and TLR9 in Coexpression with HLA-DR on CD14* ^*+*^ *Monocytes*. In order to identify the potency of antigen-presenting CD14^+^ monocytes to detect bacterial toxins, we measured the surface expression of TLR2, TLR4, and TLR9 in coexpression with HLA-DR on mature CD14^+^ monocytes.

**Figure 5:**
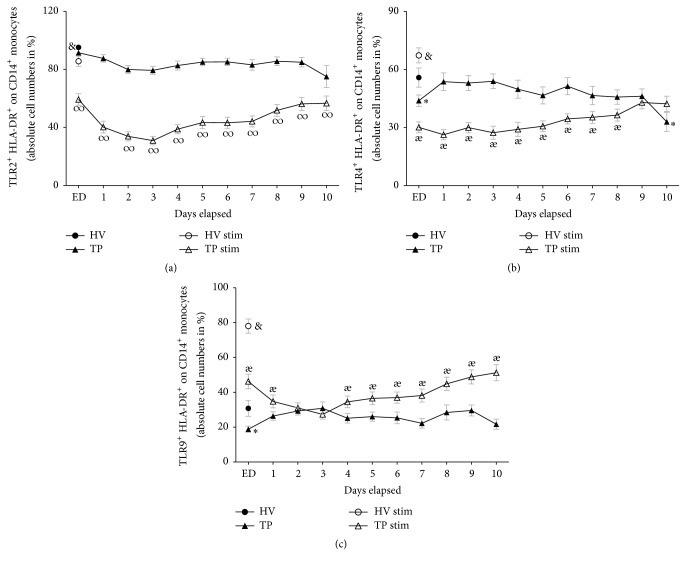
Whole blood from healthy volunteers (HV, *n* = 14) or major trauma patients (TP, *n* = 29) was analyzed by flow cytometry over a 10-day time course after admission (emergency department, ED–10). Monocytes were detected using anti-human CD14 in the corresponding sideward and forward scatter. Unstimulated (black symbols) and stimulated (clear symbols) measurements were made. For stimulation (stim), whole blood was incubated with leukocyte activation cocktail for 5 h with subsequent analyzing procedure as in unstimulated samples. Data are shown as mean ± SEM. (a) TLR2/HLA-DR coexpression on CD14^+^ monocytes, (b) TLR4/HLA-DR coexpression on CD14^+^ monocytes, and (c) TLR9/HLA-DR coexpression on CD14^+^ monocytes. ^*∗*^*p* < 0.05 versus unstimulated HV; ^*æ*^*p* < 0.05 TP versus corresponding TP stim; ^&^*p* < 0.05 versus all; ^*∞*^*p* < 0.05 TP stim versus corresponding unstimulated TP and HV stim.

During the whole posttraumatic observational period of ten days, the TLR2 and HLA-DR coexpression was significantly reduced in TP compared to HV (HV: 95.2% versus ED; 91.4 ± 1.5, *p* < 0.05, Figure 5(a)). 55.8% of all CD14^+^ monocytes from HV coexpressed TLR4^+^ HLA-DR^+^ and 30.8% of all CD14^+^ monocytes from HV coexpressed TLR9 HLA-DR. Both TLR4 HLA-DR coexpression and TLR9 HLA-DR coexpression were significantly reduced in ED. Then, they rose markedly in TP at day 1 compared to samples from ED and reached comparable coexpression levels to HV in the further posttraumatic course (TLR4 and HLA-DR coexpression ED: 43.9 ± 2.9, Figure 5(b); TLR9 and HLA-DR coexpression ED: 18.8 ± 1.7Figure 5(c)). After LAC stimulation, the HLA-DR coexpression of all measured TLRs remained at significantly lower levels in TP during the whole study period compared to stimulated samples from HV (Figures 5(a)–5(c)).

## 4. Discussion

Impaired capability of monocytes to release proinflammatory cytokines upon a secondary ex vivo-in vitro endotoxin exposure, a phenomenon termed as endotoxin tolerance, has been described in several studies for septic and trauma patients [9, 10, 36, 43, 44]. In line with these findings, we show that stimulating trauma patient's blood samples with LPS diminished the synthesis of IL-1*β* during the observation period of ten days. Interestingly, a recovery of the IL-1*β*-release upon stimulation began at day 5. The increased ratio of monocytes to leukocytes at postinjury days 6 and 7 may be one possible explanation for the observed IL-1*β* recovery. Other studies reporting a monocytosis starting at day 5 after trauma confirm our data [13]. After a secondary stimulation with LAC, CD14 expression was comparable in TP and HV. This result indicates that LAC stimulation appears rather specific to PRRs and HLA-DR, but not to CD14.

Severe trauma causes an immune dysfunction with subsequently elevated risk for multiorgan failure and infectious complications [45, 46]. Monocytes, which play a pivotal role in inflammation, show a lack in phagocytosis [35], decreased HLA-DR expression [15, 16], and an impaired cytokine secretion after an ex vivo stimulation with endotoxin in TP [9, 10, 36]. Despite numerous studies in the last decades, the detailed mechanisms of endotoxin tolerance are still not fully described [47–49]. The initial trigger for the production of proinflammatory cytokines is the signal transduction of LPS* via* TLR4, which appears on the cell surface [25, 50]. Thus, considering endotoxin tolerance, it seems reasonable to doubt the expression profile of different TLRs on monocytes after trauma. Attempts to correlate the TLR2 and TLR4 expression with the diminished immune activity on monocytes after severe trauma delivered inconsistent results [34–37]. However, these studies demonstrated an impaired function of these cells with a reduced proinflammatory cytokine release upon TLR4 stimulation [34–37]. Our data confirm the reduced monocyte activity upon a secondary stimulation after trauma (Figure 2). In fact, these studies differ notably in methods that were applied to describe the TLRs, but they also differed with regard to the timing of acquiring blood samples or the analyzed cohort of patients. Nevertheless, considering these studies, it seems that there is no correlation between the TLR expressions with the diminished cytokine release by monocytes after trauma. It is important to keep in mind that in all these studies the TLR2 or TLR4 expression was evaluated without considering the possible changes in the TLRs (co-)expression after their secondary ex vivo-in vitro stimulation.

The present study showed that the TLR2 expression was not significantly altered in unstimulated blood samples obtained from TP. Surface expression of TLR4 and TLR9 was significantly decreased only at ED (Figures 4(a)–4(c)). In summary, analyzing the native surface expression of TLRs on monocytes from TP did not deliver an explanation for the reduced capability to release IL-1*β* in monocyte (Figure 2). Tsujimoto et al. showed an increase of TLR2 and TLR4 in septic and surgical trauma patients in comparison to a control group. However, the control group expressed higher levels of TLR2 and TLR4 after the ex vivo-in vitro LPS exposure compared to surgical and septic patients [51]. In addition, an upregulation of the TLR9 gene expression in mouse macrophages was shown after LPS stimulation [52]. While the expression levels of TLR4 and TLR9 in our study were higher, TLR2 expression was lower after stimulation. Comparing samples from TP and HV, the capability of expressing TLR2, TLR4, and TLR9 after LAC stimulation was lower in TP (Figures 4(a)–4(c)). The decreased expression of TLR2 after LAC stimulation was profound in TP. A secondary stimulation after trauma may lead to an internalization or an ejection of TLR2 from CD14^+^ monocytes. Diminished TLR2 expression in monocytes from surgical patients after their LPS exposure has been reported previously [51]. Next to the impaired release of IL-1*β*, the impaired capability of expressing TLR4 in ex vivo-in vitro simulated monocytes may be partly responsible for the well-described endotoxin tolerance in trauma patients. Considering the general context, the immunological alterations after trauma appear rather unspecific, and several mechanisms may be involved in endotoxin tolerance* via* the TLR4 pathway in monocytes [53]. To name only a few, an elevated expression of IL-1 receptor associated kinase-M (IRAK-M) mRNA, a negative regulator of intracellular TLRs signalling cascade [54], has been found in monocytes from septic and endotoxin tolerant patients [55]. Furthermore a downregulation of Nlrp1 inflammasome, which is necessary for IL-1*β* synthesis, has been described in trauma patients [11].

During the complete observational period, ex vivo-in vitro stimulated CD14^+^ monocytes obtained from TP coexpressed significantly lower levels of HLA-DR^+^TLR2^+^, HLA-DR^+^TLR4^+^, and HLA-DR^+^TLR9^+^ compared to HV. Thus, the detection of bacterial stimuli after a secondary inflammatory “hit” may be impaired in TP and cause a subsequently delayed activation of further immune cells. The evaluation of unstimulated samples uncovered impaired levels of HLA-DR^+^TLR2^+^ in TP compared to HV during the whole observation period, while the HLA-DR^+^TLR4^+^ and HLA-DR^+^TLR9^+^ coexpressions were decreased in unstimulated samples at ED compared to HV.

mHLA-DR may serve as a prognostic marker for trauma and septic patients. Lower levels of HLA-DR on the cell surface have been associated with the development of sepsis after severe trauma [17, 56]. As expected, the HLA-DR expression on monocytes from TP supported the results of previous reports [15, 36, 57]. TP expressed significantly lower levels of mHLA-DR compared to HV, even more profound after ex vivo-in vitro stimulation (Figure 3(b)). Based on these data, reduced mHLA-DR expression may contribute to the higher susceptibility to infectious or even septic conditions as well. Similar findings reported before by others in septic patients showing depressed mHLA-DR expression support this hypothesis [58, 59]. The mechanisms that lead to lowered levels of mHLA-DR in septic and trauma patients are not fully understood yet. Possibly, a transcriptional downregulation of genes required for HLA-DR expression may be responsible for its diminished expression in septic patients [58]. Other authors postulated that a partially IL-10-mediated reendocytosis of HLA-DR molecules may be responsible for its lower surface expression in patients with septic shock [59]. Another study has demonstrated that the reduced percentage of HLA-DR expression may be explained by increased absolute numbers of CD14^+^ HLA-DR^−^ monocytes [60]. CD14^+^ HLA-DR^−^ cells have been characterized as a subset of myeloid derived suppressor cells (MDSC), which have immunosuppressive characteristics [61, 62]. After major surgical trauma and in septic patients, CD14^+^ HLA-DR^−^ monocytes have been shown to be upregulated as well [63, 64]. In line with these data, we found increased levels of CD14^+^ HLA-DR^−^ cells in TP, especially after the ex vivo-in vitro stimulation. Elevated levels of CD14^+^ HLA-DR^−^ cells may partly contribute to endotoxin tolerance after trauma. It is known that severe injury and surgical trauma increase the transmission of haematopoietic progenitor cells from bone marrow [65, 66], whereas the function and ability to grow out of bone marrow in culture were suppressed after severe injury [66]. These findings and our own data indicate a lower stage of maturation of monocytes with subsequently impaired function.

There are only sparse data concerning the expression characteristics of TLRs on CD14^+^ HLA-DR^−^ cells after trauma. Here, CD14^+^ HLA-DR^−^ monocytes expressed less TLR4 and TLR9 in TP compared to HV in native samples. After stimulation, the TLR4 expression on CD14^+^ HLA-DR^−^ monocytes was lowered during the whole observational period compared to HV. Due to the observation of an increased ratio of CD14^+^ HLA-DR^−^ cells after trauma, this selected subgroup may contribute to the impaired TLR4 expression on stimulated CD14^+^ monocytes after trauma.

## 5. Key Conclusions

- Reduced IL-1*β* response after ex vivo-in vitro LPS stimulation was paralleled by an impaired TLR4 expression in stimulated monocytes obtained from TP.
- In addition, an impaired TLR2 and TLR9 expression in monocytes from TP after their secondary ex vivo-in vitro simulation compared to HV was observed. Unstimulated samples showed significant reduction of TLR4 and TLR9 directly after admission.
- HLA-DR expression was lower and even more profound by LAC stimulation of monocytes after trauma.
- Increased subgroup of CD14^+^ HLA-DR^−^ monocytes expressed lower levels of TLR4 and TLR9 after trauma. After stimulation, the TLR4 expression was lowered during the whole observational period compared to HV. Due to a possibly limited signal transduction* via* TLR4 or limited function of monocytes as determined by their lower stage of maturation, these alterations may contribute to the endotoxin tolerance in TP.
- The ratio of monocytes to leukocytes was significantly increased at days 6 and 7 after trauma. This modulation may be involved in the observed recovery of the IL-1*β* release upon LPS stimulation that began at day 5 after trauma.
- Coexpression of different TLRs and HLA-DR on stimulated monocytes from TP was impaired, an effect that may cause a delayed activation of further immune cells after bacterial stimuli.

## Abbreviations

AIS: Abbreviated injury scale
BSA: Bovine serum albumin
CARS: Compensatory anti-inflammatory response syndrome
CD: Cluster of differentiation
Ctrl: Control
DAMP: Damage-associated molecular pattern
ED: Emergency department
EDTA: Ethylenediaminetetraacetic acid
ELISA: Enzyme-linked immunosorbent assay
FCS: Fetal calf serum
HLA-DR: Human leukocyte antigen-DR
HV: Healthy volunteers
ICU: Intensive care unit
IL: Interleukin
IRAK-M: Interleukin-1 receptor associated kinase-M
ISS: Injury severity score
LAC: Leukocyte activation cocktail
LPS: Lipopolysaccharide
MARS: Mixed anti-inflammatory response syndrome
MDSC: Myeloid derived suppressor cells
MHC-II: Major histocompatibility complex class II
mHLA-DR: Monocyte human leukocyte antigen-DR
MOF: Multiorgan failures
mRNA: Messenger ribonucleic acid
PBS: Phosphate-buffered saline
PICS: Persistent inflammation, immunosuppression, and catabolism syndrome
PRR: Pattern recognition receptors
SEM: Standard error of the mean
SIRS: Systemic inflammatory response syndrome
TLR: Toll-like receptor
TNF-alpha: Tumor necrosis factor-alpha
TP: Trauma patients.

## Additional Points

*Short Summary.* Reduced LPS response after trauma was paralleled by diminished expression of TLRs and HLA-DR on monocytes compared with healthy volunteers after ex vivo-in vitro stimulation.
